# Prognostic Significance of Her-2/Neu and Ki-67 Expression in Gallbladder Carcinoma: A Clinicopathological Study Across Resectable and Advanced Stages

**DOI:** 10.7759/cureus.107854

**Published:** 2026-04-28

**Authors:** Neha Singh, Ipsita Dhal, Bhagat Singh Lali, Anuj Gupta, Swapnil Patel, Shashikant Patne, Zachariah Chowdhury, Parul Tripathi, Bipinesh Sansar

**Affiliations:** 1 Pathology, Institute of Medical Sciences, Banaras Hindu University, Varanasi, IND; 2 Oncopathology, Mahamana Pandit Madan Mohan Malaviya Cancer Centre and Homi Bhabha Cancer Hospital, Tata Memorial Centre, Homi Bhabha National Institute, Varanasi, IND; 3 Oncopathology, Tata Medical Center, Kolkata, IND; 4 Medical Oncology, Mahamana Pandit Madan Mohan Malaviya Cancer Centre and Homi Bhabha Cancer Hospital, Tata Memorial Centre, Homi Bhabha National Institute, Varanasi, IND; 5 Surgical Oncology, Upkar Hospital and Cancer Institute, Varanasi, IND; 6 Pathology, Artemis Hospital, Gurgaon, IND

**Keywords:** gall bladder carcinoma, her-2/neu, ki-67, prognostic biomarker, tumor grade

## Abstract

Background

Gallbladder carcinomas (GBC) are aggressive biliary tract malignancies, which, as a result of advanced stage at presentation and poor outcome with standard treatment, lead to significant mortality. There is an unmet need to find prognostic and predictive biomarkers in GBC that can guide treatment escalation.

Hence, we aimed to study the expression of Her-2/Neu and Ki-67 by immunohistochemistry (IHC) in GBC and their correlation with clinicopathological parameters and survival outcomes.

Method

This retrospective study was conducted at a tertiary cancer center in North India over a period of 20 months, during which GBC cases were retrieved, and IHC for Her-2/Neu and Ki-67 was performed and analyzed.

Result

A total of 133 cases of GBC were analyzed, with a median age of 54.1 years, comprising 93 females (70%). Adenocarcinomas were the most common histology with 125 cases (94%), and most cases (n=54, 40.6%) were moderately differentiated. Positive Her-2/Neu expression was found in 30 cases (22.6%), with a significant loss of Her-2/Neu expression as tumor grade increased (p-value <0.05). The Ki-67 proliferation index showed a significant association with increasing tumor grade (p-value <0.05). A significant correlation was noted between higher tumor grades 2/3 and metastatic/inoperable disease, liver, and lymph node metastasis (p-value <0.05). Median overall survival (OS) was 6 months in resectable and three months in metastatic disease. However, no significant association was noted between OS and Her-2/Neu positivity, grade, or Ki-67 in metastatic/inoperable disease. In resectable GBC, median OS was significantly better in grade 1 cancers than in higher-grade 2/3 cancers by 12 months (p-value <0.05), whereas a trend towards better OS was seen in cancers with Ki-67 less than 50. Her-2/Neu positivity made no difference in OS for resectable cancers.

Conclusion

Her-2/Neu positivity declines with poor tumor differentiation, without any effect on prognosis in resectable GBC. The Ki67 index, along with tumor grading, offers potential as a predictive and prognostic marker in resectable GBC.

## Introduction

Gall bladder carcinomas (GBC) are disproportionately common in Asia, particularly in some regions of India and China. In most of these cases, they are diagnosed at an advanced stage where curative surgical treatment is not possible. Even in patients with resectable disease, the prognosis drops precipitously from stage 1 to 4. In advanced stages, the current standard of care remains a combination of chemotherapy with immunotherapy, which, however, provides only a modest survival of 12-13 months [1,2]. 

Human epidermal growth factor 2 (HER-2) expression is a known poor prognostic factor in early, locally advanced, and metastatic breast carcinomas, which has led to widespread testing for HER-2 with subsequent development of HER-2-directed therapies. Remarkably, the overall survival in these patients has improved significantly by targeting and inhibiting HER-2 expression. This has served as a key interest in finding the role of HER-2 as a major driving force of oncogenesis in other cancers, such as gastrointestinal, salivary gland, and urothelial carcinomas as well [3]. 

Among gastrointestinal tract cancers, HER-2 expression was found to be associated with poor prognosis in advanced gastric cancer, with subsequent anti-HER-2 targeted treatment approval for the same indication. A similar dismal prognosis has been noted in advanced biliary tract carcinomas (BTC) with HER-2 positivity, and several phase 3 trials are ongoing to gauge the magnitude of the benefit of HER-2 targeting agents in these patients. The effect of HER-2 expression in resectable BTC remains controversial, with some studies suggesting a poor prognosis while others denote a good prognosis. GBC is known to be different from other BTC in terms of risk factors, biology, molecular profile, gene expression, and prognosis. GBC, in addition, behaves more aggressively and has a poor prognosis compared to other BTC sub-sites. HER-2 positivity has been found to be higher in GBCs and may be one of the factors responsible for such dismal outcomes [4,5].

Ki-67, a marker of tumor proliferation, has been studied in various cancers like breast, gastrointestinal stromal tumors, and neuroendocrine tumors, among others, with a higher Ki-67 value associated with poor prognosis. Similar data for gastrointestinal tract adenocarcinomas are sparse; a potential relation to prognosis has been proposed. Morphological grading of GBC indicates differentiation, and when co-analyzed with Ki-67 values, it offers an integrated peek into the pathobiological attributes of the tumor [6-8].

Our primary objective was to correlate various clinico-pathological factors with HER-2 and Ki-67 expression, and the secondary objective was to assess their effect on long-term outcomes of both resectable and advanced GBCs.

## Materials and methods

This was a retrospective observational study at a tertiary cancer center in a high-prevalence GBC region of North India. All GBC cases that met the inclusion and exclusion criteria from May 2018 to December 2019 were included in the study.

Inclusion and exclusion criteria

We included all the patients undergoing treatment and diagnosed with GBC, whose biopsy/excision tissues in the form of paraffin blocks were available for review and immunohistochemical (IHC) analysis. 

Patients whose biopsy was inadequate, not representative of the lesion, or whose tissue was exhausted for IHC evaluation, along with all the patients lost to follow-up before treatment initiation, were excluded from the study.

All the cases of GBC were retrieved from the pathology department archives. The relevant details regarding age, gender, presenting symptoms, radiological investigations, relapse/recurrence, and survival were collected from the patient’s electronic medical records (EMR). 

The corresponding biopsy or excision specimen’s paraffin blocks and slides were retrieved, reviewed for the histomorphological details, and subjected to IHC analysis. Histopathological grading of the tumor was done depending on the gland formation, as well-differentiated (grade 1), more than 95% of the tumor composed of glands; moderately differentiated (grade 2), 50-95% gland formation; and poorly differentiated (grade 3), less than 50% of the tumor showing gland formation.

Formalin-fixed paraffin-embedded tissues were used for IHC staining. The sections were taken on pre-charged slides, and HER-2 (rabbit monoclonal antibody, clone 4B5) and Ki-67 (rabbit monoclonal antibody, clone Mib-1) IHC were performed on the representative block, using the fully automated IHC staining machine Ventana. Diamino benzidine (DAB) was used as the detection reagent. 

Invasive ductal carcinoma of the breast, already known to have positive HER-2 staining (i.e., strong and complete membranous staining in more than 10% of the tumor cells, Score 3+), and lymph nodes were taken as positive controls for HER2 and Ki-67, respectively. For analysis of HER-2 IHC, interpretation of HER-2 was done as per the American Society of Clinical Oncology/College of American Pathologists protocol for gastric cancer, wherein a score of 3+ (positive) was intense membranous, complete, or basolateral/lateral staining in ≥ 10% of tumor cells in a resected specimen or ≥ 5 tumor cell clusters on biopsy. Scores 0 and 1+ were negative, where score 0 was absence or membranous staining in < 10% tumor cells in surgical specimens, and score 1+ was very faint/barely perceptible membranous staining in ≥ 10% tumor cells in surgical specimens or incomplete membranous staining in ≥ 5-cell clusters in biopsy. Score 2+ was equivocal, with weak to moderate membranous staining (complete or basolateral/lateral) in ≥ 10% of tumor cells in surgical specimens or in ≥ 5 tumor cell clusters in biopsy specimens [9]. Due to the unavailability of reflex fluorescence in situ hybridization (FISH) testing in our study, a score of 2+ was taken as negative.

The Ki-67 proliferation index was calculated by analyzing the percentage of cells showing nuclear positivity after counting one thousand cells in the highest proliferation zone of the tumor. Ki-67 was divided into low proliferation (<50%) and high proliferation (>50%) categories.

Overall survival (OS) was defined as the time from diagnosis to death from any cause. Progression-free survival (PFS) was calculated from initiation of systemic therapy to radiological or clinical progression or death, whichever occurred first; patients without an event were censored at last contact.

The collected data was tabulated and analyzed using statistical tests for the distribution of data using IBM Corp. Released 2024. IBM SPSS Statistics for Windows, Version 30. Armonk, NY: IBM Corp. These tests included proportions, mean, median, mode, Fisher’s exact test (two-tailed), and chi-square test. Survival estimates were calculated by the Kaplan-Meier method using the log-rank test, and a p-value of <0.05 was considered statistically significant. Median follow-up duration was calculated by the reverse Kaplan-Meier method.

The study was approved by the Institutional Ethics Committee with a waiver of written informed consent due to its retrospective design. All procedures adhered to the ethical principles of the Declaration of Helsinki (2013 revision), and the study was conducted and reported in accordance with the Strengthening the Reporting of Observational Studies in Epidemiology (STROBE) guidelines.

## Results

**Figure 1:**
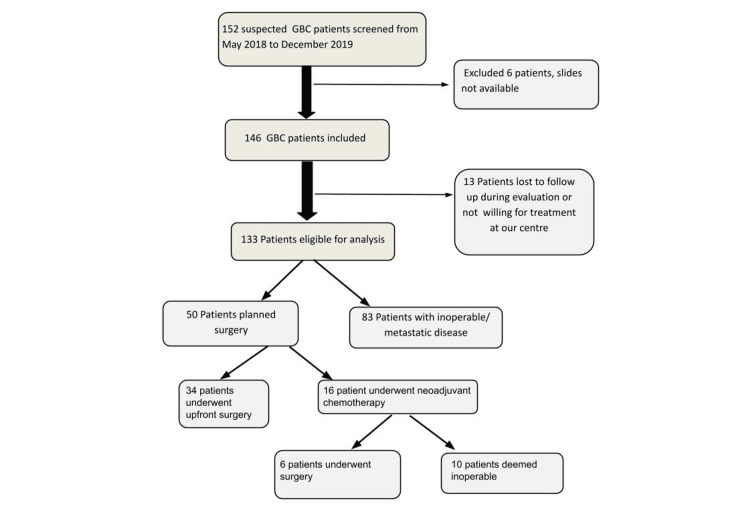
Consort diagram GBC: Gall bladder carcinoma

We have depicted the selected 133 patients for analysis in the Consort diagram (Figure 1).

**Figure 2:**
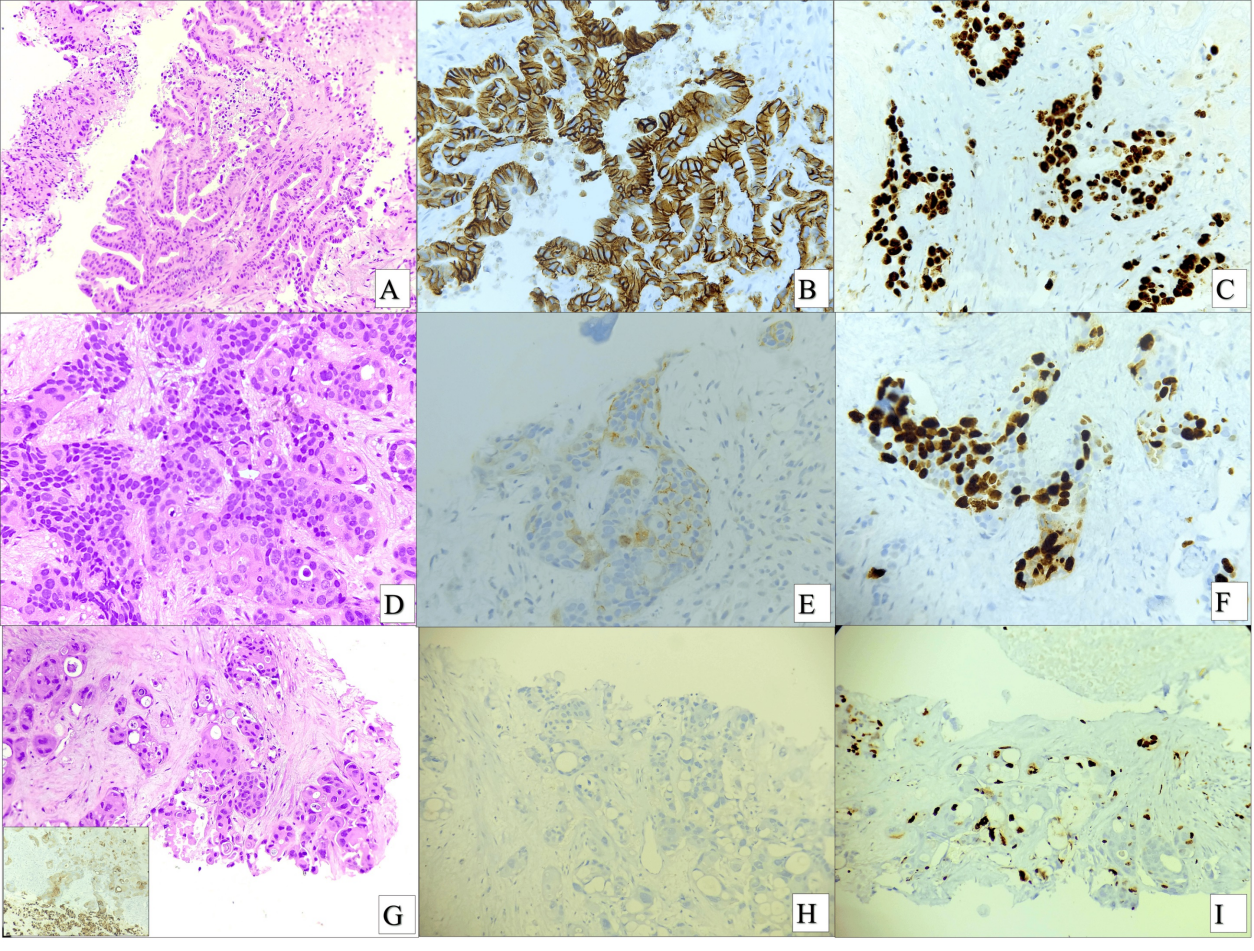
Histopathology and immunohistochemistry expression in gallbladder carcinomas. A. Section shows a well-differentiated adenocarcinoma (HE, 4X) with B. positive (score 3+). Her2/Neu IHC stain. C. and a high Ki-67 proliferation index (approximately 70%) D. Poorly differentiated adenocarcinoma (HE, 10X) E. with a Her2/Neu score of 2+ by IHC and F. Ki-67 proliferation index of approximately 50-60%. G. Poorly differentiated carcinoma (HE, 4X), inset showing CK7 positivity, with H. negative (score 0) Her2/Neu, and I. Ki-67 proliferation index of approximately 40%. HE: Hematoxylin and Eosin; IHC: immunohistochemistry; CK: Cytokeratin

The median age was 54.1 years (range: 21 to 80 years) with a female predominance (93 cases, 70%). The most common histology was adenocarcinoma (125 cases, 94%), followed by adeno-squamous carcinoma (5 cases, 3.76%) and one each (0.75%) of intra-cystic papillary carcinoma, mucin-secreting adenocarcinoma, and signet ring cell adenocarcinoma. Morphologically, 54 cases (40.6%) were moderately differentiated (grade 2), followed by 46 cases (34.6%) of well-differentiated (grade 1) and 33 cases (24.8%) of poorly differentiated cases (grade 3) (Figure 2).

While studying the distribution of tumor grade, we noted that higher grades 2/3 were significantly associated with metastatic/inoperable disease (p-value = 0.01). Similarly, a significant correlation was noted with respect to liver metastasis (p-value= 0.02), along with lymph node metastasis (p-value= 0.03), suggesting that higher grades have a greater tendency to metastasize. 

Positive HER-2 expression (score 3+) was found in 30 cases (22.6%), while 77 cases (57.9%) were negative (score 0 to 1). Twenty-six cases (19.5%) had an IHC score of 2+. The percentage positivity of HER-2 in stage I, II, III, and IV was 16.7% (1 case), 10.5% (2 cases), 35.3% (12 cases), and 20.3% (15 cases), respectively. The correlation between HER-2 expression and various clinico-pathological features, along with their significance, is shown in Table 1.

**Table 1:** Her2/Neu and Ki-67 staining results with respect to various study parameters Statistical analysis: Chi-square test was used when expected cell counts were ≥ 5. *Fisher’s exact test was used when expected cell counts were <5. *Fisher’s exact test does not produce a test statistic value; only the p-value is given. HER2: Her2-Neu, G: Grade, χ2: Chi-square

Variable	Category	HER2 positive	HER2 negative	Ki-67 High	Ki-67 Low	Grade 1	Grade 2/3	Total
Gender	Female	21(22.6%)	72(77.4%)	68(73.1%)	25(25.9%)	34(36.6%)	59(63.4%)	93
	Male	9(22.5%)	31(77.5%)	33(82.1%)	7(17.9%)	13(32.5%)	27(67.5%)	40
	χ^2^, p value	0.004, 0.95		1.194, 0.28		0.202, 0.02		
Grade	G1	14(30.4%)	32(69.6%)	29(63%)	17(37%)	_	_	46
	G2	13(24%)	41(76%)	43(79.6%)	11(20.4%)	_	_	54
	G3	4(12.1%)	29(87.9%)	29(87.9%)	4(12.1%)	_	_	33
	χ^2^, p value	3.67, 0.16		7.04, 0.03				
Stage	1	1(16.7%)	5(83.3%)	5(83.3%)	1(16.7%)	1(16.7%)	5(87.3%)	6
	2	2(10.5%)	17(89.5%)	12(63.2%)	7(36.8%)	10(52.6%)	9(47.4%)	19
	3	12(35.3%)	22(64.7%)	26(76.4%)	8(33.6%)	14(41.2%)	20(59.8%)	34
	4	15(20.3%)	59(79.7%)	57(77%)	17(23%)	22(29.7%)	52(70.3%)	74
	p value*	0.18*		0.58*		0.16*		
Metastatic / Inoperable disease	Yes	16 (19.3%)	67(80.7%)	66 (79.5%)	17(20.5%)	22(26.5%)	61(73.5%)	83
	No	14 (28%)	36 (72%)	34 (68%)	16 (32%)	24(48%)	26(52%)	50
	χ^2^, p value	1.274, 0.26		2.637, 0.1		6.132, 0.01		
Liver Metastases	Yes	13(21%)	49(79%)	47(75.80%)	15(24.20%)	16(25.8%)	46(74.2%)	62
	No	18(25.4%)	53(74.6%)	54(76%)	17(24%)	31(43.7%)	40(46.3%)	71
	χ^2^, p value	0.603, 0.44		0.007, 0.93		5.256, 0.02		
Lymph nodal metastases	Yes	11(20.4%)	43(79.6%)	41(75.9%)	13(24.1%)	13(24%)	41(76%)	54
	No	20(25.3%)	59(74.7%)	61(77.2%)	18(22.8%)	33(41.8%)	46(58.2%)	79
	χ^2^, p value	0.473, 0.49		0, 1		4.489, 0.03		
Ki-67 Status	High	21(20.8%)	79(78.2%)	—	—	30(29.7%)	71(70.3%)	101
	Low	9(28.1%)	23(71.9%)	—	—	17(53.1%)	15(46.9%)	32
	χ^2^, p value	0.701, 0.40				6.214, 0.01		

HER-2 expression was significantly lost from grade 1 (14 cases) to grade 3 (4 cases), which was significant (p value <0.05) (Table 2).

**Table 2:** Her2/Neu expression and tumor grade. Statistical analysis: Fisher’s exact test was used. A test statistical value is not produced; only the p-value is given. HER2: Her2-Neu, G: Grade

HER2 Score	G1	G2	G3	Total
0	12(20.33%)	25(42.37%)	22(37.28%)	59
1+	6(33.33%)	9(50%)	3(16.67%)	18
2+	14(53.84%)	8(30.76%)	4(15.38%)	26
3+	14(46.66%)	12(40%)	4(13.33%)	30
Total	46(34.59%)	54(40.60%)	33(24.81%)	133
P value - 0.02				

The Ki-67 proliferation index ranged from 5% to 99% in the tumors (median 70%), which increased with increasing grade. 

We noted 83 cases (62.4%) that were metastatic or inoperable at the time of presentation, of which 61 cases (73.5%) were of grade 2 or grade 3, which was significant (p value=0.01). However, no significant correlation was noted with metastasis or inoperable disease with HER2 positivity or increasing Ki-67 index (Table 1).

Of the 34 patients undergoing upfront radical cholecystectomy, 28 patients received adjuvant chemotherapy. Three patients of stage 1 were kept on observation, whereas 3 patients opted out of adjuvant therapy. The chemotherapy regimen in patients undergoing adjuvant or neoadjuvant, or first-line palliative chemotherapy was a combination of gemcitabine and cisplatin or oxaliplatin, except for some stage 2 patients who received adjuvant capecitabine alone. Among the patients with inoperable/advanced disease, only 39/83 (47%) took palliative chemotherapy; the rest were planned for best supportive care.

**Figure 3:**
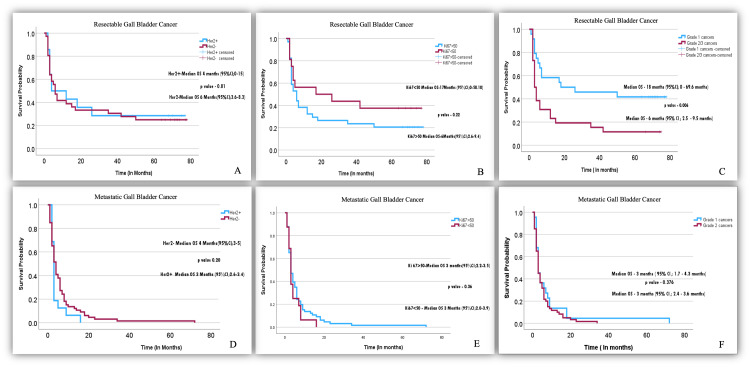
Overall survival in resectable and metastatic disease. A-C: Difference in overall survival with respect to Her2/Neu positivity, high Ki-67, and grade 1 versus higher grades in resectable diseases, respectively. D-F: Difference in overall survival with respect to Her2/Neu positivity, high Ki-67, and grade 1 versus higher grades in metastatic diseases, respectively. Her2: Her-2/Neu, OS: Overall Survival, CI: Confidence Interval

The median follow-up was 72 months (95% confidence interval (CI): 68.6 to 75.4 months). The overall survival was six months (95% CI: 2.5 to 9.4 months) in resectable GBC and three months (95% CI: 2.3 to 3.7 months) in the metastatic setting. Comparative OS between different groups in resectable and metastatic disease is shown in Figure 3. The median progression-free survival of patients undergoing palliative chemotherapy was three months (95% CI: 2.3 to 3.7 months). Median overall survival in patients undergoing palliative chemotherapy was five months (95% CI: 3.4 to 6.6 months).

At the end of follow-up, all the patients with metastatic disease had died (83/83, 100%); however, 13 patients were alive in the resectable group (13/50, 26%). Four patients with resectable GBC were lost to follow-up after adjuvant treatment and were censored.

## Discussion

We aimed to analyze the expression of HER-2 positivity and Ki-67 proliferation across various clinico-pathological parameters in resectable and metastatic gall bladder carcinomas and to find out their correlation with overall survival. 

Our study had a similar distribution of age, gender, and histomorphological profile as several previous studies. We also noted maximum HER-2 positivity in well-differentiated tumors (14 cases, 46.6%) compared to poorly differentiated tumors (4 cases, 13.3%), with a significant correlation, a finding that was similarly noted in previous studies [10,11]. 

Our data suggested a maximum HER-2 positivity in stage 3 cancers (12 cases, 35.3%), followed by stage 4 cancers (15 cases, 20.3%), while it remained low in stages 1 and 2. A similar trend was noted by Chen et al. from China, with the difference being lower HER-2 positivity of stage 2 cancers in our study compared to 30% in their study. This could be a result of the relatively smaller number of stage 2 cases in our study cohort. Few studies have reported the distribution of HER-2 positivity with other poor prognostic factors, like lymph nodal and distant metastasis. However, we noted no difference across HER-2 positive and negative spectrums [12]. 

The overall survival difference in resectable GBC with HER-2 positivity was not statistically significant in the whole resectable group, and also when compared by stage. Even in the group of metastatic GBC, there was no difference in the overall survival between HER-2-positive and negative groups. This might have occurred, probably as none of the patients received HER-2-directed therapy, which, at the time of study, was not the standard. Recent evidence points to a benefit in progression-free survival and overall survival with the addition of HER-2-directed therapies like trastuzumab, zanidatamab, and trastuzumab-deruxtecan in metastatic GBC, which leads us to conclude that although HER-2 positivity might not always be a prognostic marker, it can definitely be considered as a predictive biomarker [12-14].

Our results appear similar to those seen in gastric cancers, where the role of HER-2 positivity has been studied in greater detail and indicates a poor prognosis in metastatic disease, with improved outcomes with HER-2-directed therapy. However, the efficacy of HER-2-directed therapy in improving outcomes in the adjuvant setting appears to have failed in gastric cancer, and hence it can be hypothesized that HER-2-positive GBC may follow a similar trajectory in the curative setting [15,16]. 

Ki-67, another interesting marker studied extensively in breast carcinomas, correlates with prognosis, with higher expression indicating a more aggressive disease. In our study, we used a Ki-67 index of 50% and above to indicate a high Ki-67 status, similar to some studies, apart from breast cancer, where a higher cut-off of 40 or more was preferred. We observed that GBC with a low Ki-67 index could be metastatic, whereas those with a high Ki-67 index could still be in the early stages. While the correlation of Ki-67 to tumour grade was expected, it was interesting to note that this was not a linear relation [6,7,11].

Overall survival was numerically much higher in the resectable GBC group with low Ki-67 compared to the high Ki-67 group, with a difference of around 11 months, although this was not statistically significant. Since nearly all patients in both groups had received similar adjuvant treatment, we suggest a strong consideration for research on escalating adjuvant treatment in patients with higher Ki-67. In the metastatic group, there was no difference in survival with Ki-67 grouping, probably indicating a poor chemosensitivity of GBC in general [17].

Grade of tumor is a known prognostic factor in gastrointestinal cancers, but has not been correlated extensively in GBC. We observed important positive findings with respect to grading and good outcomes in GBC. Grade 1 GBC was more likely to be resectable with less chance of lymph nodal and liver metastases. Overall survival was significantly better in resectable GBC with grade 1 tumors compared to higher grades. However, in the metastatic setting, no difference was observed with respect to grade, suggesting a potentially complex association determining the disease tempo [6,18]. 

An important limitation of our study is that we could not do reflex FISH testing for HER-2 in cases where IHC was scored 2+ due to logistical issues. As FISH testing might have reclassified 20-50% of these patients as HER-2 positive, our findings could have changed [9]. Other limitations included the retrospective nature of the study, single-institute data, and a significantly lower number of patients receiving chemotherapy for metastatic disease due to the COVID-19 pandemic during the study period, which may have potentially affected our observations on the influence of treatment outcomes by introducing treatment heterogeneity.

The strength of our study is that our institute lies in the region of the highest GBC incidence in our country, and it reflects the pattern of GBC biology. Furthermore, we have a robust median follow-up of six years for survival data.

## Conclusions

HER-2 testing has evolved into an integral part of the diagnostic algorithm for GBC, which, to date, remains difficult to treat. Our data captures their biological behavior and prognosis across the spectrum of disease, suggesting their limited role in the adjuvant setting. Ki-67, a promising biomarker with potential to guide treatment escalation in the adjuvant setting, should be studied in further detail to extract potential practice-changing observations. Well-differentiated adenocarcinomas (grade 1) have good outcomes, whereas higher-grade GBCs merit adjuvant treatment escalation trials. These biomarkers should be studied in new trials, tailoring treatment in metastatic and resectable settings to usher in improvements and a new era of precision and personalized medicine.

## References

[1] Rawla P, Sunkara T, Thandra KC, Barsouk A (2019). Epidemiology of gallbladder cancer. Clin Exp Hepatol.

[2] Zhou Y, Yuan K, Yang Y (2023). Gallbladder cancer: current and future treatment options. Front Pharmacol.

[3] Goddard KA, Weinmann S, Richert-Boe K (2012). HER2 evaluation and its impact on breast cancer treatment decisions. Public Health Genomics.

[4] Abrahao-Machado LF, Scapulatempo-Neto C (2016). HER2 testing in gastric cancer: An update. World J Gastroenterol.

[5] Ayasun R, Ozer M, Sahin I (2023). The role of HER2 status in the biliary tract cancers. Cancers (Basel).

[6] Davey MG, Hynes SO, Kerin MJ (2021). Ki-67 as a prognostic biomarker in invasive breast cancer. Cancers (Basel).

[7] Gupta A, Gupta S, Rajput D (2021). Expression and clinicopathological correlation of Ki-67 in gallbladder carcinoma. J Carcinog.

[8] Hidalgo Grau LA, Badia JM, Salvador CA (2004). Gallbladder carcinoma: the role of p53 protein overexpression and Ki-67 antigen expression as prognostic markers. HPB (Oxford).

[9] Bartley AN, Washington MK, Ventura CB (2016). HER2 testing and clinical decision making in gastroesophageal adenocarcinoma: guideline from the College of American Pathologists, American Society for Clinical Pathology, and American Society of Clinical Oncology. Am J Clin Pathol.

[10] Dash S, Anirvan P, Samantaray S (2025). Human epidermal growth factor receptor-2/neu expression in gallbladder cancer is significantly associated with clinicopathological parameters and survival. Indian J Gastroenterol.

[11] Halder S, Kundu S, Chakraborty J, Chakrabarti S (2019). Significance of HER2 and Ki-67 in preneoplastic lesions and carcinoma of gallbladder. J Gastrointest Cancer.

[12] Chen L, Xu L, Shen L (2021). HER2 positivity is affected by the papillary structure and has a bidirectional prognostic value for gallbladder carcinoma. Front Genet.

[13] Ostwal V, Mandavkar S, Bhargava P (2024). Trastuzumab plus gemcitabine-cisplatin for treatment-naïve human epidermal growth factor receptor 2-positive biliary tract adenocarcinoma: a multicenter, open-label, phase II study (TAB). J Clin Oncol.

[14] Theocharopoulos C, Ziogas IA, Mungo B (2025). HER2-targeted therapies: unraveling their role in biliary tract cancers. Crit Rev Oncol Hematol.

[15] Yoshida H, Shimada K, Kosuge T, Hiraoka N (2016). A significant subgroup of resectable gallbladder cancer patients has an HER2 positive status. Virchows Arch.

[16] Lago NM, Villar MV, Ponte RV (2020). Impact of HER2 status in resected gastric or gastroesophageal junction adenocarcinoma in a Western population. Ecancermedicalscience.

[17] Kashyap L, Singh A, Tomar S (2023). Pattern of care and outcomes of gallbladder cancer patients: retrospective study from a high incidence region in India. South Asian J Cancer.

[18] Tran T, Ethun CG, Pawlik TM (2017). Histologic classification and grading enhances gallbladder cancer staging: A population-based prognostic score validated by the U.S. Extrahepatic Biliary Malignancy Consortium. J Clin Oncol.

